# Supporting the Biomedical Science UG Project Research Journey Through Staff-Student Partnerships

**DOI:** 10.3389/bjbs.2024.12215

**Published:** 2024-05-29

**Authors:** S. Veuger, L. Cookson, H. Creighton, S. Gallaher, S. Racey, M. Ridley, I. Robson

**Affiliations:** ^1^ Department of Applied Sciences, Northumbria University, Newcastle upon Tyne, United Kingdom; ^2^ Department of Social Work, Education and Community Wellbeing, Northumbria University, Newcastle upon Tyne, United Kingdom

**Keywords:** biomedical science, students as partners, research project, employability, graduate attributes

## Abstract

**Introduction:** Developing research skills enhances graduate attributes and student employability. The UG research project is coined the pedagogy of the 21st century but the diversity of supervisory styles is a source of student perceived inequality of experience. The goal of this study was to provide structure and support to undergraduate (UG) biomedical science research students and supervisors by co-creating research informed resources that are accessible, engaging and student centred. We asked 1) How do UG students experience research supervision? 2) What approaches do supervisors use to support UG project students? 3) How do students as partners benefit from being involved in pedagogical research?

**Materials and Methods:** In Stage One, 3 UG student research partners co-developed questionnaires and followed these up with semi-structured interviews. Fifty two UG project students took part in an interactive poll and 14 supervisors answered a questionnaire. Seven students and 4 supervisors were interviewed. These were analysed by thematic analysis. In Stage Two, the questions were asked of UG project students (*n* = 79) via an interactive poll and the resource developed in Stage One was trialled with students (*n* = 68) and supervisors (*n* = 37).

**Results:** The global theme identified was that students feel strongly that the student-supervisor relationship influences their experience, satisfaction and success. In all polls, >90% of students but <60% of supervisors agree that a good student/supervisor partnership has an effect on the success of the final project. A smaller percentage of students felt strongly that they were able to develop a successful partnership with their supervisor. We co-created a visual model and a list of discussion points of how the student-supervisor partnership can be developed, aimed at making supervision more effective whilst being non-prescriptive.

**Discussion:** The resource can be easily adapted. Students believe it helped them to develop a staff-student partnership and supervisors commented that it helps to clarify roles and manage student expectations. This scalable project will support the practice of future UG biomedical science project research students and supervisors. Working with students as partners enabled the development of richer ideas whilst supporting their employability.

## Introduction

Working in partnership with students as partners (SaP) holds immense value in ensuring that we uncover concerns and develop proactive responses that impact those who are directly affected [1]. In doing so, we are able to shift the focus from staff to students to develop authentic student centred resources, fostering student engagement and driving increased student responsibility for their learning. Partnership can take many forms as discussed by Healey et al and works best when it forms part of the culture and ethos of a department or institution [15]. Partnership with students is therefore effective when a sense of community can be built amongst staff and students. This project sought to work with students to develop resources that drive the development of a culture and community of individual staff-student research partners. In doing so, the study seeks to support the biomedical science undergraduate (UG) project research journey, increasing both the graduate attributes and employability of biomedical science students.

Employability can be considered a set of knowledge, skills and behaviours that support university graduates to be successful in their chosen career path [2]. Employers are seeking graduates with high level transferrable skills alongside personal attributes. Programmes accredited by the Institute for Biomedical Sciences (IBMS) seek to ensure that students receive wide ranging research-informed scientific education and develop skills and experience that employers value. In addition to running and developing new tests, biomedical scientists undertake research and therefore benchmark statements for biomedical sciences include the ability to execute independent research-centred data generation, analyse, interpret and critically evaluate data [3]. As many as 34% of biomedical science graduates choose to enter research as a career [4]. Important skills for researchers are broad ranging and include analytical, communication, problem solving, data analysis, critical thinking and team working. These skills enable the development of solutions to complex problems and therefore research skills are highly valued by employers as they are essential to a wide range of industries.

Graduate attributes and the final destination of Higher Education (HE) leavers both impact university ranking. There are sector-wide concerns over students’ career readiness and difficulties transitioning from university into a working environment [5]. Employability is therefore a key Teaching Excellence Framework (TEF) metric and embedding opportunities to support the development of skills into the UG curriculum can positively enhance graduate employability. Pedagogic approaches to developing students as independent researchers that optimise the development of research skills are therefore beneficial to the development of student employability.

The UG research project is the most sustained research heavy piece of work that students undertake during their degree programme. Coined the pedagogy of the twenty-first century [6,7], numerous studies have reported research as a pedagogic practice [8–10]. Serbic and Bourne identify the final year research project as a tool for maximising the employability prospects of students [10]. During their final year UG research project, students are expected to review the literature, collect and analyse data and write up independently. The shift from tutor-directed to self-directed learning is often cited as a mechanism to drive independent learning in final year UG research project students [11]. Students are able to make explicit links between taught material and knowledge with professional applications. The UG research project is therefore an ideal mechanism to develop students as researchers [12] whilst encouraging a deep approach to learning and fostering employability skills.

Anecdotally, supervisors distinguish supervision from other forms of teaching, viewing the UG research project as a unique opportunity for the student to venture into a new territory where authority and relationships are reconfigured [13]. The UG research project is an important learning experience at the end of the biomedical science (BMS) programme. Uniquely, this is delivered by multiple members of staff which leads to diverse approaches to the supervision of projects. This range of supervisory styles is perceived by students as an inequality in experience. The focus that is taken by the supervisor during the research project can vary and also change throughout the project and may not rely on only one approach. Pedagogic research-teaching approaches defined by Healey et al [15] include “research-led” (learning about current research in a discipline), “research-oriented” (developing research skills and techniques), “research-tutored” (engaging in research discussions) and “research-based” approaches (undertaking research and inquiry) [15].

The BSc. (Hons) biomedical sciences programme at Northumbria University recruits in the region of 200 students per year. A major consideration for proactive students is the employability aspects of their curriculum vitae (CV) following a degree programme. Student feedback also highlights a belief that there is a benefit from opportunities to engage in learning and assessment activities that help them develop and enhance their employability. The programme at Northumbria is accredited by the IBMS. As well as an expectation that students will undertake independent research, the IBMS benchmark statements highlight that there should be “a commitment to equity and inclusive practices for diverse student cohorts through considered course design” [3].

Firmly aimed at enhancing the student learning experience, graduate attributes and employability, the aims of this action research project were to 1) improve supervision quality to allow all students studying biomedical science to achieve their potential and realise their ambitions, irrespective of their background or motivations for studying biomedical Science. 2) work in partnership with students to co-create a robust solution that “values and harnesses differences and encourages openness and participation where everyone feels respected, supported and valued” [3].

Our goal was not to change the subject specific aspects of the UG research project but to develop mechanisms that support the pedagogical teaching approach. The IBMS benchmark also states that “students should expect to be embraced as partners within their own courses ….. student voice should play a significant role in course development, delivery, review and the overall student experience within biomedical science” [3].

The objectives of this action research project were 1) to work with students as partners (SaP) to understand the perceptions and expectations of students and supervisors of the UG research project 2) to understand the developing identity of UG research project students as researchers 3) with inclusivity and partnership in mind, to co-create research informed resources that are accessible, engaging and student-centred 4) to reflexively assess the benefits of participating in pedagogical research for the UG student co-researchers 5) to trial and evaluate a “making supervision work” resource with biomedical science supervisors and their students.

## Methodology

**FIGURE 1:**
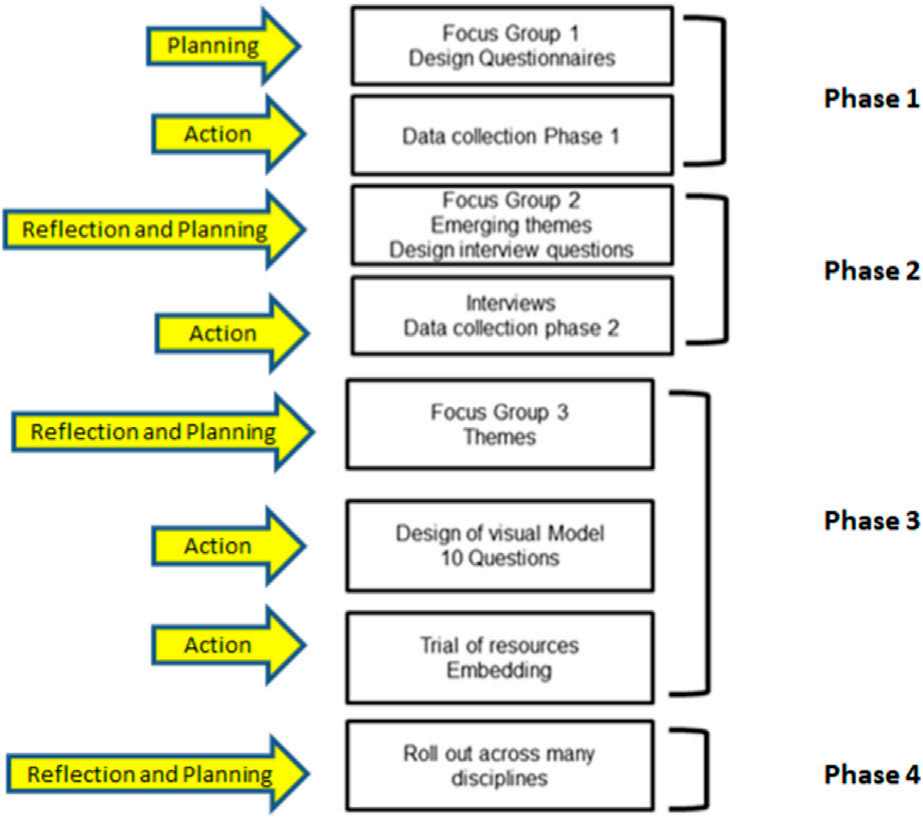
Phases of this action research project. Stage One of the study followed three key phases incorporating UG student co-researcher training, question design, data collection and theme development [14]. Stage Two of the study has finalised phase 3 by trialling the resource in the programme of biomedical science and started phase 4 of rolling out the study into other disciplines.

This study is qualitative, participatory, small-scale pedagogical research. It is an interpretive project with a focus on understanding the subjective experience and process of UG biomedical science research. This action research project is sustainable and ongoing since 2016 with 4 phases undertaken in two key stages (Figure 1). Stage One involved initial data collection and resource development [14], and Stage Two involved further data collection and trial of the resource. The study was and continues to be informed by the concept of working in partnership with students [15] as change agents. In this study, UG students are involved in the scholarship of teaching and learning [16]. In working with UG students as equal partners, the project was participatory and aspects of the project design were co-designed with students. In Stage One, 3 UG student co-researchers were involved in all aspects; study design, methods, resource development and dissemination of the outputs. In Stage Two, 2 biomedical science UG project students collected further data on the experience of students using the questionnaire designed in Stage One (Supplementary S1). They also developed a supervisor student feedback sheet and collected narrative responses from students and supervisors on the resource developed in Stage One (Supplementary S4).

In Stage One, 3 selected methods (questionnaires, semi-structured interviews and focus groups) were used [17]. Stage Two of the study utilised questionnaires only. Biomedical science UG students were co-researchers, collecting data for their own UG research project. We used the POWER framework described by Verwood and Smith (2020) to ensure all students felt empowered to fully contribute [18].

Stage One of the study followed three key phases incorporating UG student co-researcher training, question design, data collection and theme development [14].

Stage Two of the study has finalised phase 3 by trialling the resource in the programme of biomedical science and started phase 4 of rolling out the study into other disciplines.

### Participants

Participants were identified through a combination of purposive and convenience sampling. In Stage One, all UG students *n* = 111 (44 Males, 67 Females) including the UG student co-researchers (*n* = 3) were enrolled on the final year 40 credit UG research project of the biomedical science programme at Northumbria University and were invited to participate in this study through questionnaire and interview (Supplementary S1, S3). All supervisory staff (*n* = 67) were invited to complete a questionnaire and then invited for a follow up interview (Supplementary S2, S4). In Stage Two, all UG students *n* = 158 (73 Males, 85 Females) were enrolled on the final year 40 credit UG biomedical science research project and were invited to participate in an interactive poll (Supplementary S1). All supervisory staff *n* = 67 and students *n* = 158 were invited to trial the resource and provide their qualitative perceptions via feedback questionnaire (Supplementary S4).

Recruitment was via a central email. There was no solicitation of volunteers. It was made clear that participation is voluntary and a full participant information sheet (PIS) was provided. The process of consent included opportunity for questions about the research to be raised.

### Focus Groups

Focus groups were run both as an initial training exercise for the student co-researchers involved with the research and as a mechanism to work together to design questions and draw out themes from the data. For both Stage One and Two, focus groups ensured we worked as a collective research team in equal partnership for all aspects of the study. In Stage One, the detail of the methods and how they were implemented were co-designed with the 3 UG student co-researchers. In Stage Two, the focus groups enabled, the co-creation of the questionnaire to evaluate the resource (Supplementary S4).

### Questionnaires

UG biomedical science students *n* = 52 (Stage One 46% response rate) and *n* = 79 (Stage Two 50% response rate) anonymously completed a questionnaire containing 16 questions (Supplementary S1) as an interactive poll. Using a five point Likert scale, the student questionnaire provided students with the opportunity to reflect on and respond quantitatively about their perceptions and expectations of the UG research project alongside their researcher identity.

Supervisors *n* = 14 (20% response rate) were given a similar questionnaire in Stage One (Supplementary S2) via email which also incorporated opportunity for free text responses to qualitatively consider their supervision style, views on the benefit of research as well as exploring aspects of the student-supervisor partnership.

In Stage Two, supervisors *n* = 37 (55% response rate) and their UG biomedical science research project students *n* = 68 (43% response rate) trialled the resource and filled a questionnaire (Supplementary S4).

### Interviews

The data from the questionnaires in Stage One enabled us to begin early theme development and to explore these themes via semi-structured interviews to encourage dialogue. Broad themes of confidence, independence and the importance of the supervisor-student partnership were identified in the questionnaire data. Interview questions were written with the 3 UG student co-researchers to explore these aspects further (Supplementary S3).

In Stage One, four supervisor (3 Males, 1 Female) and seven student (2 Males, 5 Females) interviews took place. All interviewees had previously filled in the questionnaire. The interviews were carried out by the 3 UG student co-researchers who emphasised a) that the process is appreciative, so they are to think about “what worked” and what would be “even better if,” and, b) the purpose of the reflective activity is to appreciate their experience and insights.

### Data Analysis

The data from both questionnaires and interviews were analysed and discussed in focus groups with the whole research team. Thematic analysis looked at the perceptions and experiences of supervision for both students and supervisors [14].

We used a mixed methods approach with elements of qualitative and quantitative methods [19]. Quantitative data focussed on median Likert scores whilst analysis of qualitative narratives from interviews and free text in questionnaire responses were used to evaluate the perceptions and expectations of both students and supervisors of the UG research project. Analysis of open-ended responses to interviews took a grounded approach. In addition to data in the form of transcripts of audio-recorded interviews, the project generated reflective and reflexive data. Transcripts were subject to basic coding analysis to generate themes for further reflection and group (academic staff and student co-researcher) discussion. This two-step analysis therefore built on initial themes. The interviews were analysed individually, informed by a phenomenological approach to qualitative data [14].

## Results

All polls and interviews show that supervisors and students consider the UG research project to be a valuable experience. However, they also showed that the diversity of supervisory styles is a source of student perceived inequality of experience for UG biomedical science research project students.

**FIGURE 2:**
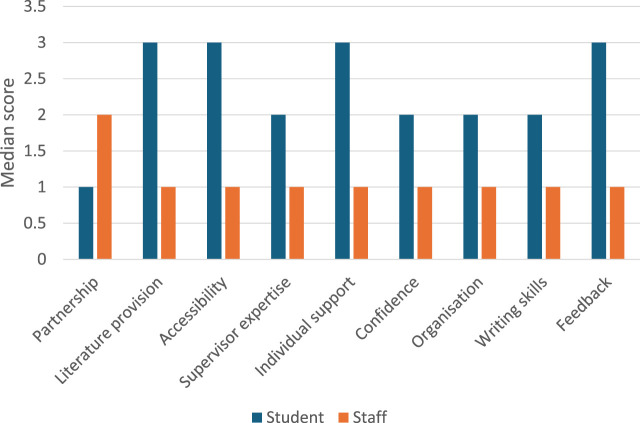
Alignment of student and supervisor views in the Stage One poll. Perceptions and expectations of aspects of supervision for students (*n* = 52) and supervisors (*n* = 14) as ranked using the median scores of a Likert scale. 1 = strongly agree, 2 = agree, 3 = neutral, 4 = disagree, 5 = strongly disagree. Data derived using questionnaire responses in Stage One. Questions 1–9 relate to each of the nine themes shown for students and supervisors (see Supplementary S1, S2).

Selected questions from the Stage One questionnaires were compared for their median scores to see if there are areas where scores and comments align or show disparity (Figure 2). A complex picture emerged about the students’ expectations for their UG research project and the student-supervisor partnership in comparison to what the supervisors agreed to be important. Although student views were not fully consistent with supervisors (for literature provision, accessibility, individual support and feedback), there are some interesting areas of overlap with respect to partnership, staff expertise, student confidence, organisation and writing skills.

**FIGURE 3:**
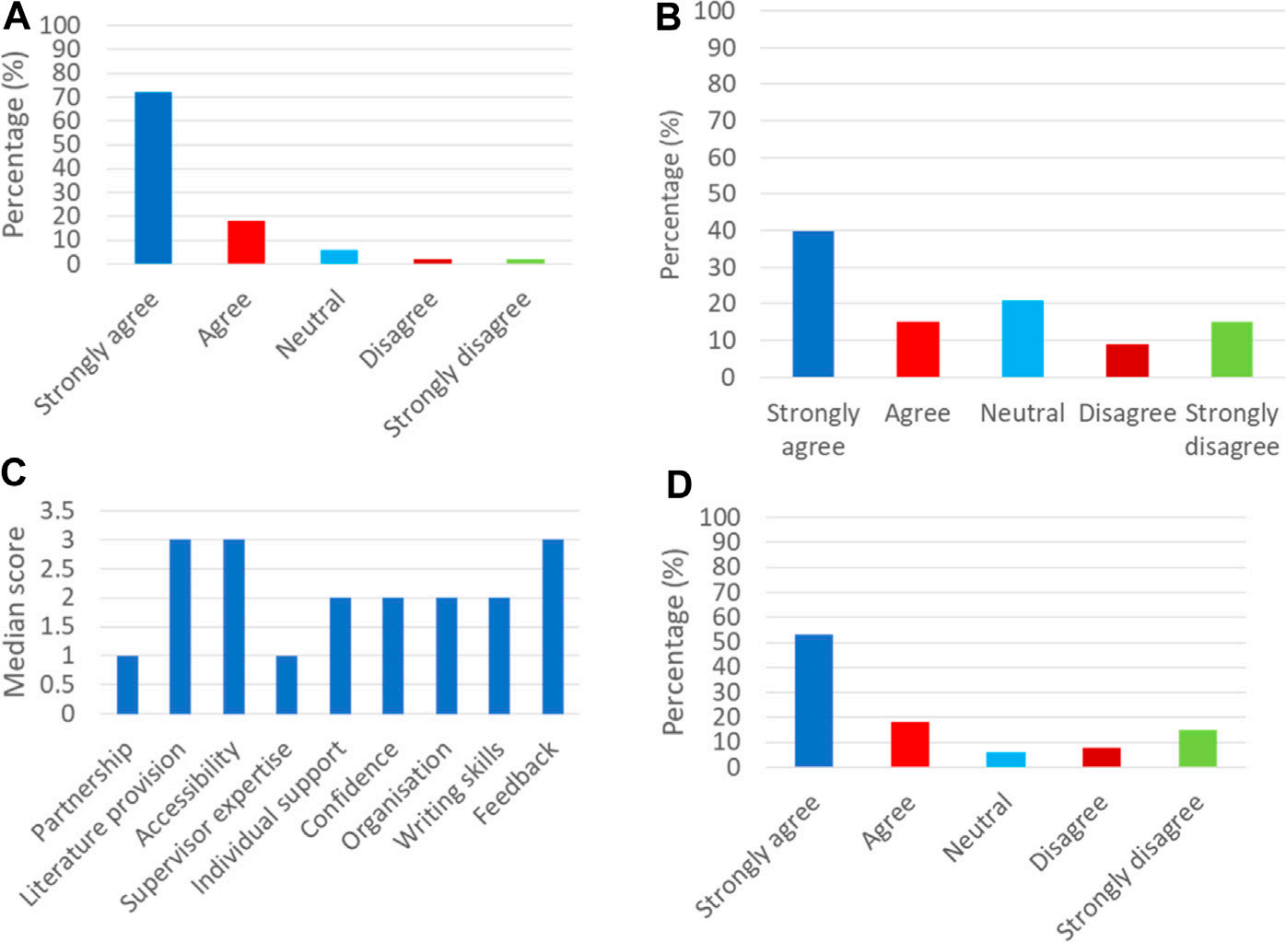
Student perceptions of their project success. **(A)** Stage One poll (*n* = 52): Q1 90% students believe that the student-supervisor partnership strongly influences the success of their project. **(B)** Stage One poll (*n* = 52): Q11, only 40% of students strongly agreed that they were able to build a strong partnership with their supervisor. **(C)** Student poll Stage Two (*n* = 79). Perceptions and expectations of aspects of supervision for students as ranked using the median scores of a Likert scale. 1 = strongly agree, 2 = agree, 3 = neutral, 4 = disagree, 5 = strongly disagree. Data derived using interactive poll responses in Stage Two. Questions 1–9 relate to each of the nine themes shown for students (see Supplementary S1). **(D)** Stage Two poll (*n* = 79): Q 11, 53% of students strongly agree they were able to build a strong partnership with their supervisor.

The global theme identified from the student data was that students feel strongly that the student-supervisor relationship influences their experience, satisfaction and success. Across all questionnaires in both Stage One and 2, >90% of students (versus <60% of staff) strongly agreed/agree that the student-supervisor partnership influences the success of their UG research project (Q1 Supplementary S1, S2, Stage Two student data shown in Figure 3A). Whilst none of the supervisors said they strongly disagreed with this statement 29% were neutral (*n* = 4, Q1 Supplementary S2). Strikingly, only 40% of students strongly agreed that they felt they had achieved a partnership with their supervisor in the Stage One questionnaires (*n* = 21, Q11 Supplementary S1, Figure 3B). Moreover, 15% of students strongly disagreed which indicates varied practice amongst supervisors (*n* = 7, Figure 3B). Interestingly, the median Likert score of three for students being able to build a strong partnership mirrored that of the staff belief about its importance (neutral, Q11 Supplementary S1 and Q13 Supplementary S2). In Stage Two, the student interactive poll (Figure 3C) showed similar patterns to the poll in Stage One shown in Figure 2. Interestingly students were more favourable in rating individualised support with the median improving from 3 (neutral) to 2 (agree) (Figure 3C) and the percentage of students who strongly agreed that they had achieved a partnership rose to 53% (*n* = 42, Figure 3D) which may be positively influenced by the Stage Two small scale trial of the resource developed in Stage One. However, not all respondents to the Stage Two poll had trialled the resource (68 trialled the resource and 79/158 students answered the poll) and therefore a true quantitative measure using these scores for the impact of the resource is not able to be drawn.

The researcher identities of students had developed as a result of the UG research project experience (>70% strongly agreed/agreed across both questionnaires; Stage One, *n* = 40 and Stage Two, *n* = 61. Q6 Supplementary S1) and the majority of supervisors (>80%, *n* = 12) strongly agreed/agreed that students had grown in confidence, becoming more organised and independent (Q6, Supplementary S2). Similarly, >65% of students strongly agreed/agreed that they had become more organised and independent (*n* = 35, Q7, Supplementary S1). Greater than 50% of students disagreed or strongly disagreed that they were confidant before the project (*n* = 27, Q13, Supplementary S1) whilst >80% agreed the experience of undertaking research had increased their confidence (*n* = 46, Q14, Supplementary S1). However, perceptions around the student-supervisor partnership were mixed, with supervisors not necessarily attributing the increase in student confidence and acquisition of skills to the development of a partnership, preferring to state that the process of the research was responsible for this increase (*n* = 4, Q13, Supplementary S2). More than 50% of supervisors were neutral or disagreed that they should be directive (*n* = 12, Q14, Supplementary S2) whilst 90% state that working independently is important (*n* = 13, Q11, Supplementary S2).

These findings were explored further in Stage One interviews with questions focussed on understanding what worked and what could have been done differently. Student and supervisor voice showed some similar ideas around the importance and perception of a partnership. Students commented that feeling they were working in a partnership helps build trust and the confidence to ask questions which in turn helped to make them feel more supported, driving their motivation.

“Need to be able to have a good relationship to be able to ask questions and advice.” (UG project student)

Moreover, others reported that a partnership with their supervisor promoted feelings of belonging and acceptance through approachability and respect that in turn drives independence.

“I felt there was mutual respect and that my supervisor had confidence in me to allow me to work independently.” (UG project student)

Staff views varied with some believing the partnership to not be relevant or adding the caveat that this is about raising awareness to the student that independence is expected.

“The partnership is important as long as it’s the student that owns the project.” (supervisor)

Others take a more student-centred approach, believing their approach should be about what works for the individual student

“It depends on the needs of each student—everyone is different.” (supervisor)

Fostering independence is an important element of the UG research project and staff are keen to ensure that students are independent. Students commented during interviews that the reason they lacked confidence was because this was a new endeavour and that this led them to question their capability to conduct independent research. Issues for students included anxiety, isolation and lacking a clear structure.

“Taking responsibility for my own learning creates uncertainty.” (UG project student).

“We were given instructions and left to work independently without constant supervision. At times this was scary.” (UG project student)

Students commented that through partnership with their supervisor, these concerns could be allayed. Staff and students agree that the supervisor role changes as students move through project with staff discussing the need to strike a balance and scaffold their support:

“It is important they try to think of solutions themselves. It is an autonomous module.” (supervisor)

“Some direction at the start of project is needed but after that the majority of direction should be self-direction.” (supervisor)

The majority of the students interviewed said that the primary role of the supervisor is to give support. Whilst >80% of both students (*n* = 42) and supervisors (*n* = 14) strongly agreed/agreed in the Stage One poll that the member of staff were sufficiently skilled to guide the research (Q4 Supplementary S1, S2). Most comments from students were related to aspects of flexibility, approachability and support (Figure 2), highlighting the variety of approaches offered by different supervisors. The same trends were seen in the follow up student poll in Stage Two (Figure 3C). The ease with which students felt they could meet with their supervisor varied, with 25% of students across both Stage One and 2 polls strongly disagreeing that they were able to do this when required whilst staff believed they made efforts to be accessible to their students (Figure 2 median Likert scores 3 neutral versus 1 strongly agree. Q3 Supplementary S1, S2). In addition, students reflected that a good student-supervisor relationship should ensure that individual preferences and needs are considered and supported accordingly. Greater than 50% (*n* = 9) of supervisors strongly agreed in the Stage One poll that they provide individualised support, whilst none disagreed (Q5 Supplementary S2). However, >40% of students (*n* = 23) in the Stage One poll were neutral or disagreed that their supervisor appreciated their individual needs (Q5 Supplementary S1) although this improved slightly in the Stage Two poll with 52% agreeing (*n* = 41). The attempt by supervisors at balancing provision of support to drive the move towards autonomy may result in students feeling less supported. The differences may also reflect the emphasis placed on the type of support.

Three organising themes were identified in the Stage One interviews; education support, practical support and emotional support. Narratives highlight that a difference in the type of support offered may account for the different perceptions of students and staff. Supervisors placed more emphasis on supporting practical and educational skills whilst many students spoke at length about emotional support.

“My personal struggles impact my ability to do well at university. My supervisor was not interested in this which made the project hard.” (UG project student)

Supervisors focussed ons skills development (including communication and time management skills) while students believe strongly that to ensure that a good working relationship is developed, staff need to engage in emotional support. This will improve researcher confidence and help with stress.

“I advised on their practical ability to help them gain the best mark possible.” (supervisor)

“I emailed protocols each week so that everything was planned ahead of time.” (supervisor)

Interestingly, whilst supervisors focussed on skills, they were less inclined to support writing skills:

“No, these are not research skills and should’ve been learned and developed at previous levels/modules and APPLIED in the project. Students need guidance on format and not writing skills.” (supervisor)

In terms of practical support, students expressed frustration at the lack of communication and flexibility of their supervisor and differences between resources provided by supervisors. The provision of literature was one area where there was mixed practice which is perceived by students as a disparity that was driving their dissatisfaction.

“My friend was provided with 5 key papers but my supervisor said that was not their role.” (UG project student)

“Providing a key paper only but must not give the students their literature search.” (supervisor)

“This is critical so they have an understanding of previous literature that underpins work.” (supervisor)

Students felt that educational support is required to provide direction and motivation for the topic area. Although there is agreement that feedback is an important mechanism to drive learning and reflection in students, >30% of students across both the Stage One (*n* = 19) and Stage Two (*n* = 24) questionnaires disagreed that feedback was helpful or constructive (Median score 3, neutral. Figures 2, 3C, Q9 Supplementary S1). In contrast, > 50% of staff strongly agree (*n* = 8) that they provide quality feedback (Median score 1, strongly agree. Figure 2, Q9 Supplementary S2).

“Marking of drafts was integral on the feedback process along with discussion each week. On the occasion when students had not submitted drafts the report mark was affected.” (supervisor)

“Verbal feedback is an important part of the supervision meetings.” (supervisor)

Perhaps students do not fully recognise the value of verbal discussions as feedback:

“It is unfortunate that our conversations are poorly recalled by students during write up.” (supervisor)

Students commented positively on the skills that they developed as a result of carrying out a piece of research and importantly >65% (*n* = 35) of students strongly agreed/agreed (>30% remained neutral or disagreed, *n* = 17) that they were more likely to choose research as a destination demonstrating a developing identity as a researcher (Stage One poll, Q15, Supplementary S1). Greater than 60% (*n* = 32) of students said they had improved their critical thinking and writing skills (Stage One poll, Q8, Supplementary S1).

“I learned to critically evaluate sources and I have a much better understanding of how to arrive at a good piece of work” (UG project student)

However, some staff are focussed on the research outputs rather than the learning of the students:

“The students should already have these skills.” (supervisor)

Interestingly some staff commented that a good student-supervisor relationship as having an impact on the developing researcher identities of UG project research students whilst others felt it was the research process itself.

“The working relationship is important so there are open and frank discussions between student and supervisor. This allows them to develop as researchers.” (supervisor)

### Resource Development—Parity Without Prescription

The first few meetings that a supervisor has with each student are therefore critical and can help to set the tone for the whole research experience. Supervisors were clear that they did not want resources that direct them to supervise in a prescriptive manner with 50% *n* = 7 voting neutral or disagreeing (Q10 Supplementary S2). Nevertheless, the perceived disparity in experience that students talk about means that there needs to be a mechanism whereby students feel they are receiving a parity of experience. The results have shown that this can be achieved by ensuring the following conditions are met:

• There should be an open discussion between each staff -student pair at the beginning of the project. This should contextualise the project and a discussion to agree how they will build a positive working partnership.
• Expectations of both student and supervisor should be clarified.
• Setting of realistic targets for each person throughout the project.
• Regular communication and flexibility with respect to resources (e.g., literature provision).
• Opportunities to ask questions should be provided.
• Ability for students to negotiate the style of supervision they receive.

**FIGURE 4:**
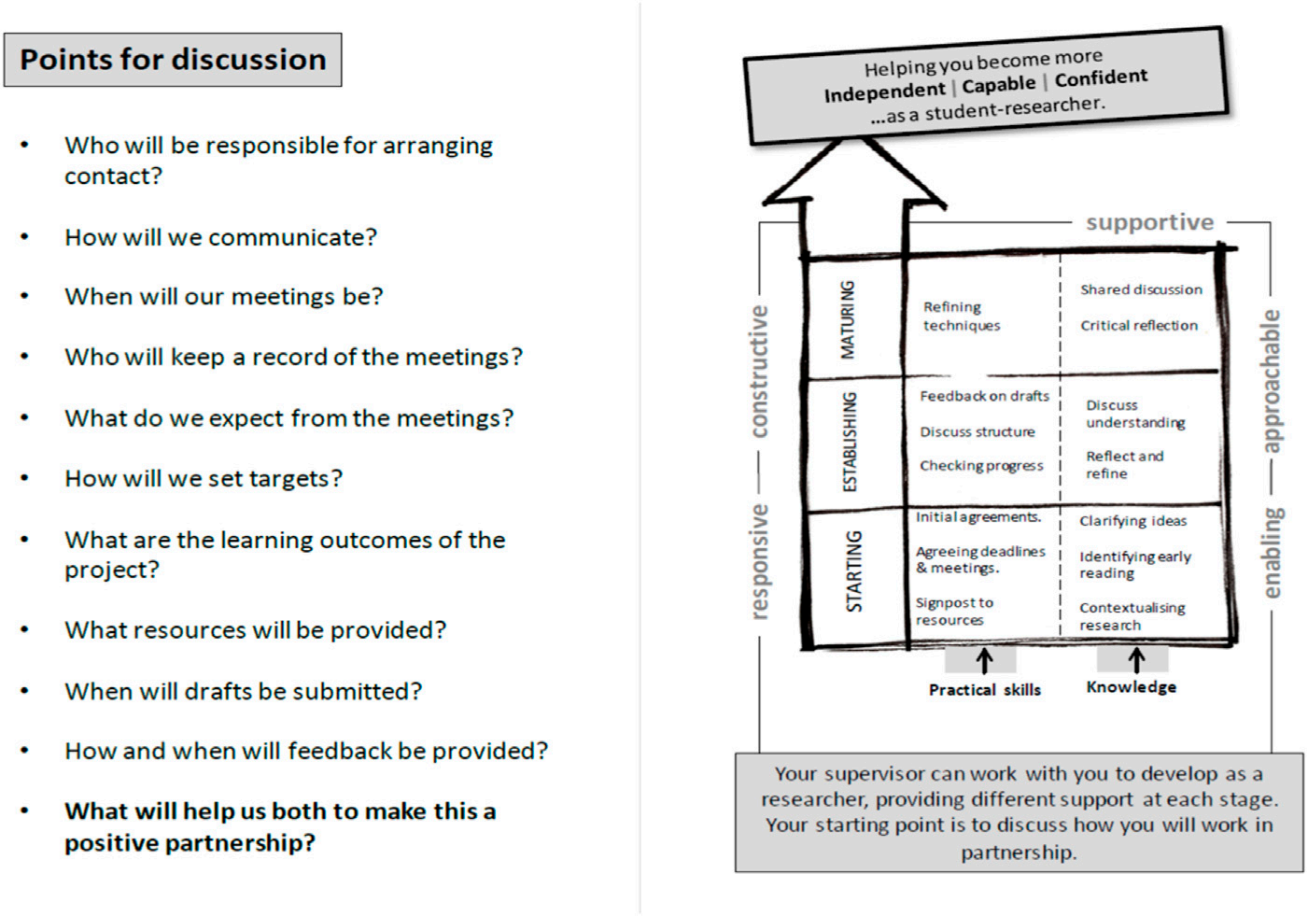
Making supervision work resource developed in Stage One and trialled in Stage Two. 10 questions to discuss between each supervisor and student at an initial 1:1 meeting. Visual model highlighting types of support and the changing nature of supervision. Resource was co-developed as a research team with 3 UG students. Design by author Iain Robson [14].

With the 3 UG student co-researchers, we co-created a “making supervision work” resource in Stage One (Figure 4) to help support students and supervisors manage their expectations and ensure that the opportunities in the above wish list are provided.

### Evaluation

The resource shown in Figure 4 was evaluated in collaboration with 2 UG project researchers in Stage Two of the study. Feedback from supervisors and students was positive, highlighting that using the 10 questions alongside the visual model at the first meeting was mutually beneficial for all concerned and served to increase student confidence. An important finding was that 86% (*n* = 59) of students who trialled the resource commented that the points for discussion supported the development of the student supervisor partnership (Qs 6 and 7, Supplementary S4). Encouragingly, all supervisors with the exception of one said they would use the resource in future rounds of supervision (verbal feedback on collection of feedback sheets and follow up).

“I believe I did so much better thanks to the ability to talk through the supervisory approach because I felt much less anxious.” (UG project student Q7 Supplementary S4)

“Being able to feel free to ask lots of questions has made me feel a lot less worried.” (UG project student Q7 Supplementary S4)

Supervisors were able to take a student-centred approach:

“Very valuable in clarifying roles and managing student expectations.” (Supervisor Q5 Supplementary S4)

For other supervisors, it enabled them to support students to develop independence and ownership of the research while recognising that they need to build in some flexibility.

“The exercise clarified the responsibilities of the students - that this was a piece of independent research work, and the role of the supervisor is to guide them through the journey rather than telling them what to do.” (Supervisor Q2, Supplementary S4)

Supervisors commented that agreement on two particular questions (“Who will be responsible for arranging contact” and “Who will keep a record of meetings”) will drive motivation and support the shift towards independence (Q3, Supplementary S4)

### Benefits of Working in Partnership With Students as Partners

In the Stage One focus group 3, the 3 UG student project co-researchers were asked to reflexively consider the benefits of their involvement in this study.

Student co-researchers unanimously feel that they are more employable having developed/refined a range of desirable skills that they would not have developed through the course alone; including leadership skills, professionalism, communication skills and decision making skills.

“Being able to work with staff and students as peers to carry out the project was greatly beneficial and increased confidence in my ability to research and communicate.” (Stage One UG student project co-researcher)

“I personally got a huge boost in confidence. It has given me an insight into working with other people on a professional manner which is a great thing to take away.” (Stage One UG student project co-researcher)

Working in partnership gave students a sense of identity and improved their confidence.

“Working with staff as an equal partner gave me confidence to think like a researcher.” (Stage One UG student project co-researcher)

In Stage Two of the project, one of the UG project students who was also a co-researcher commented that they were able to rethink theoretical concepts and practical issues in ways they had not considered before.

“As a biomedical science student, qualitative research is very new to me ….I have learned more research skills than I thought I ever would.” (Stage Two, UG student project co-researcher)

## Discussion

Graduates in biomedical Sciences will develop the qualities of “professionalism, critical independent thought, and decision-making in complex and unpredictable circumstances” [3]. Jenkins and Healey argue “all UG students in all HE institutions should experience learning through and about research and inquiry” [20]. The UG research project is considered a journey towards independent thought achieved through a shift in focus from tutor-directed to self-directed learning. It needs to offer the best possible experience to students to ensure they are able to develop the skills set required to engage and progress into employment. Here, we describe our approach to working with students as partners (SaP) to develop resources that support the student-supervisor partnership to ensure we offer our biomedical science students an UG research project experience that is inclusive and harnesses differences. One that encourages “openness and participation so that everyone feels respected, supported and valued” [3] and moves away from considering the project as a purely research endeavour. As previously described [14], Healey, Flint and Harrington’s conceptual framework helped us to locate our research as “co-researching and co-inquiring,” somewhere in the overlap between “subject based research” and “scholarship of learning and teaching” [15]. This study investigated the inclusion of students in subject-based inquiry, as well as the scholarship of teaching and learning where students engage in pedagogical research. Therefore, students were not only involved in carrying out research towards their final UG research project, but engaged alongside staff in pedagogical research into the student research experience. All students involved as co-researchers throughout both stages of the project felt they had developed important skills that enhance their employability. Working in partnership with staff has gained recognition in HE, recently being described as a “powerful approach to enhancing the quality of education and fostering more engaging and inclusive learning environments” [21]. We found that actively including students as co-researchers and developers in this study drove positive learning experiences and a sense of developing professionalism for the students whilst staff benefitted from discussion with those that the research was directly impacting. In doing so, student researchers not only brought newer ideas that had not been previously considered but were also able to garner richer more honest feedback from their peers that in turn supported the development and evaluation of an inclusive and equitable resource.

This study built on previous work [14], further demonstrating that there are differing expectations between students and staff and this drives dissatisfaction amongst students particularly as the supervision approaches vary. Each supervision is unique and is affected by a number of factors including the student -supervisor relationship. This study has highlighted that the approaches and views of supervisors can vary greatly. Moreover, negotiation, motivation, ability to ask questions, skills development, communication and the changing nature of supervision over time were all concerns of both supervisors and students.

Many of the supervisors interviewed said that supervision is one of their most enjoyable but challenging aspects of their role, citing the development of students into independent researchers as a significant achievement. Particular benefits of the UG research project include the promotion of critical thinking, increased confidence and the intention to pursue post-graduate research [22]. For UG biomedical science students there is also the benefit of developing key laboratory skills as well as their analytical skills. In this study, UG biomedical science research project students reported that they also learned many general skills not necessarily all science based to include literature searching and referencing skills. Supervision approaches can vary considerably which may impact the student experience. Research projects undertaken by students on our biomedical science programme are diverse involving mainly wet lab projects but also dry projects including bioinformatics and systematic reviews. All projects require 120 h of research and analysis and although this data is not available for this study, it would be interesting to investigate whether the type of project influences student satisfaction and success. Both students and supervisors recognised the value of the individual supervisor for their knowledge and expertise. Students and supervisors also agreed that students grow in confidence and were able to develop skills including writing and organisation. Although supervisors were focused on ensuring students developed excellent practical skills through a range of approaches, supervisors were less willing to provide emotional support, perhaps because they feel they lack the expertise or they do not consider this to be part of their role. Providing a mechanism (Figure 4) where supervisors and students can openly discuss this aspect of supervision is therefore very valuable even if it means supervisors choose to refer students for further support elsewhere. Moreover, the resource helps to define the respective roles of the student and supervisor, helping to drive independence and a researcher identity in UG biomedical science students.

As a starting point—the use of the resource (Figure 4) enables each student-supervisor pair to have a discussion at the start of the project. The 10 questions serve as prompts to help focus the discussion. The key issue is that students feel they can raise any concerns and ask questions. The answers are personal to each supervisor/student pair but the use of a standard set of questions provides parity, clarity and structure. The visual model highlights the changes in power as the project progresses from the supervisor as tutor through mentor through to peer. It also enables a discussion that explores the types of support that will be provided. This is especially useful where there is a variety in approach amongst supervisors/mismatches in expectations between student and supervisor (e.g., provision of literature). An open discussion allows each person to explain their viewpoints and enable an understanding to be reached for each student supervisee pair rather than a broad set of directives that staff may see as too directive.

Our findings show that the challenge is to strike a balance in the level of support provided in order to support a move towards independence. This can be difficult to do and many supervisors grapple with the need to support their students whilst also leaving them to puzzle concepts for themselves [23]. Del Rio et al explored this more recently and their findings are in line with ours, suggesting that supervision involves a complex interaction between autonomy and support [24]. In our study, a lack of accessibility was frequently cited by students as a factor that drives dissatisfaction along with being left too much on their own. Yet, students also acknowledged that being left to think through problems for themselves drove their independence.

“At times I felt really alone …looking back—I learned a lot during those uncertain times.” (student)

Walkington (2015) conceptualised five successive levels of student involvement in their research project which were adapted to incorporate 10 dimensions of effective UG research supervision; focus, motivation, inclusivity, setting collaboration, originality, content, audience, compensation and staff-student relationship [25,26]. Each of these represents a continuum and our research across multiple years of biomedical science students shows that partnership (one end of the continuum) is preferred by students and viewed as highly beneficial, driving confidence, independence and a researcher identity as well as alleviating feelings of anxiety and uncertainty.

In this study, we have brought together multiple pedagogical frameworks - pedagogy of employability, pedagogy of the UG research project and pedagogy of SaP [1, 7, 25, 27]. We worked in equal partnership with UG biomedical science students to uncover the expectations and perceptions of students and supervisors. We used the results to develop the following outputs that seek to support the research journey and drive the development of graduate attributes of biomedical science students:

• Evidence for the importance of the student–supervisor partnership in driving confidence and researcher identity.

The importance of the student—supervisor partnership in developing a “student-researcher” pedagogy is significant as it shifts the role of the staff member as knowledge provider to that of co-inquirer, facilitating students to become experts in their research area.

• A resource to support the supervision process and aid the development of a partnership (Figure 4). This includes a visual model of how the student supervisor partnership changes over time and 10 questions to support discussions between each student–supervisor pair.

Feedback demonstrated that the resources can act as a framework to help reduce the disparity and therefore discontent felt by students as a result of perceived differences in approach taken by supervisors.

• Evidence that inclusion of students as equal partners in pedagogic research as co-enquirers and co-creators enables the development of richer more authentic resources to support the curriculum.
• Evidence that student co-researchers benefit by further developing their employability and graduate attributes.

Supervisory styles are often described as a spectrum from laissez-faire to authoritarian, with no one style fitting every situation. Supervisors should adapt their approach to accommodate the student and the stage of the research project. Moreover, this study has highlighted the importance of striking a balance between a “research focus” and a “student development focus.” Therefore, the context in which the research is taking place is important in determining the approach to take at a given stage. Our research highlighted the most effective practices of supervisors to be; responding to students’ needs and abilities throughout the research process, setting clear expectations, teaching the methods for the discipline, balancing emotional support with expectations and supporting students to take ownership of the research. Our model (Figure 4) highlights the changing nature of supervision over the course of the research project and encourages supervisors to reflect on their personal style and to further consider what would work for each supervisee pair. The findings in this study agree with Del Rio et al, who concluded that the role of the supervisor should be clarified beforehand as well as consideration of the skills to be developed and the supervisor’s position on the support that will be provided [24].

### Study Limitations

There are limitations to the approach taken including power relationships, the motivation of students to get involved, their ability and the research has been limited to one department. The numbers of students and staff who gave their views were unequal and staff views were only obtained in a poll in Stage One and not Stage Two. This makes some comparisons and therefore quantitative evaluation of the impact of the resource difficult. However, the aim was to develop resources through the process of action research. Qualitative feedback from both staff and students presented in this manuscript has been very positive and the leaflet and 10 questions therefore serve as a starting point for individual departments who can then decide how to build on these findings.

## Conclusion

This study sought to support the pedagogy of the UG research project for UG biomedical science students by uncovering the perceptions and expectations of both supervisors and students through a student as partners approach. In this study, we worked in collaboration to support the biomedical science UG research project students and supervisors by co-creating resources that are inclusive and student-centred. Supervisors indicated that approaches to explicitly guide their supervision was not favoured and would be resisted (Q10, Supplementary S2). The model and discussion points are simple whilst being non-prescriptive and can be easily adapted to the needs of different programmes (Box 1). When embedded into the programme, they represent a mechanism to support the pedagogy of employability. We believe that this scalable project will support the practice of future project students and supervisors through the development of graduates that are distinguished by their intellectual expertise and employability. Moreover, the inclusion of students as co-researchers and co-developers enables the development of resources that are inclusive and equitable as well as supporting the employability of those students.

Box 1 **Advice for Biomedical Science Supervisors Who Wish to Adopt the Resources.**

• The supervision process cannot be a prescriptive one.
• Working with students as partners (SaP) provides a robust real world application where the research activity responds directly to the needs of the participants. Consider working with students to refine the resource for your course.
• Use the resource in the first meeting between each supervisor-student pair to facilitate discussion and uncover expectations.
• The student supervisor relationship is very important and this can be developed easily by clarifying expectations for each person throughout the project. Are there other questions that you might discuss?
• Consider having students share the agreed answers to the questions and have both student and supervisor sign this.
• Encourage students to develop their metacognition by building in key points to reflect on their research using the model and refine the supervision approach if necessary.
The resource in Figure 4 is flexible and can be adapted for use in other programmes.

## Summary Table

### What Is Known About This Subject

• The biomedical science UG research project is an important high stakes assessment.
• QAA subject benchmark: a commitment inclusive practices for diverse student cohorts through considered course design.
• QAA subject benchmark: Student voice should play a significant role in the student experience within Biomedical Science.

### What This Paper Adds

•   Evidence based recommendations to enable staff to build on their supervision style.
•   The co-created model and discussion points are simple yet non-prescriptive and can be easily adapted.
•   Benefits of including students as equal partners in pedagogic research for the development of graduate attributes.

## Concluding Statement

This work represents an advance in biomedical science because the ability to carry out independent research and develop a researcher identity promotes the development of key skills that are essential for future employment.

## Data Availability

The data presented in this article are not all readily available as per our ethics approval. Requests to access the datasets should be directed to s.veuger@northumbria.ac.uk.
