# Supplementary material for: Supporting the Biomedical Science UG Project Research Journey Through Staff-Student Partnerships
Source: Br J Biomed Sci. 2024 May 29;81:12215. doi: 10.3389/bjbs.2024.12215 (PMC11167481; doi:10.3389/bjbs.2024.12215)
Supplement: Supplementary file 1 [file Table1.pdf]

S1 Student questionnaire

1 The supervisor / student partnership is an important influence on the success of the final project.
2 My supervisor has supported me by suggesting academic literature relevant to my project.
3 I found it easy to meet with my supervisor when required.
4 My supervisor was sufficiently skilled to guide me through my research project
5 I felt my supervisor appreciated my individual preferences and needs.
6 Work with my supervisor has increased my confidence as a researcher
7 As a result of working on my research project I have become more organised and independent as a researcher.
8 Conducting my research (design, data collection, analysis, writing) has improved my critical thinking and writing skills.
9 I found supervisor feedback on my work appropriate and constructive
10 Working with my supervisor has clarified my research topic and design.
11 I was able to build a strong partnership with my supervisor.
12 My supervisor was enthusiastic and encouraging about my research topic.
13 Before undertaking my research project, I understood and felt confident about what was ahead of me
14 Having experience of research has increased my confidence
15 I feel more likely to conduct research in the future following my experience in my final year project
16 I now appreciate the issues associated with conducting ethical research

S2 Supervisor Questionnaire

	Question	Strongly Agree	Agree	Neutral	Disagree	Strongly Disagree	Please Explain
1	**Building a partnership between myself and the students I supervise has an influence on the success of the final project**						
2	**It is important to support students by suggesting academic literature relevant to the project**						
3	**I make efforts to be accessible to students as and when they need my advice**						
4	**I have the skills and knowledge to confidently support students I supervise**						

**5**	**I provide individualised support to each student**						
**6**	**The students I supervise grow in their confidence as researchers**						
**7**	**I support my students to become organised and independent**						
**8**	**It is important to support the academic writing skills of the students I supervise**						
**9**	**I actively provide verbal feedback to my students during the course of their project**						
**10**	**Guidance and expectations for supervisors should be made more explicit**						
**11**	**I expect my students to be able to work independently**						
**12**	**It is important that I have subject expertise in the projects that I supervise**						
**13**	**The partnership I build with my students is critical to their academic success**						
**14**	**It is important to be directive in the support I provide to students I supervise**						

S3 Interview questions

- In the questionnaire responses, students told us that the supervisor / student relationship was important.
  - *Why do you think this is important?* [same for supervisor]
  - *Can you tell me a little more about what has worked well for you?* [same for supervisor]
- In the questionnaire, most students told us that their confidence, skills and understanding of research has developed since the beginning of their projects.
  - *What has helped you to become more independent?* [how have you supported your students to become more confident, independent and skilled as researchers?]
  - *Can you give me examples of things you can do more effectively now than before in research?*

S4 Resource feedback sheet

**PLEASE ANSWER ALL THE QUESTIONS & GIVE YOUR UG PROJECT STUDENT A COPY OF THE LEAFLET:**	
1. Did you use the leaflet in the meeting with your student?	
YES	NO
Please Explain:	
2. Did the leaflet support the conversation between you (as the Supervisor) and your Project student?	
YES	NO
Please Explain:	
3. Through using the leaflet did you make any agreements/follow up points with your Project student?	
YES	NO
Please Explain:	
4. Would you make any changes to the leaflet?	

YES	NO
Please Explain:	
5. Do you have any other comments/observations about the leaflet you would like to share?	
YES	NO
Please Explain:	
6. Please show the leaflet to your Project student: Do they understand the purpose of the leaflet?	
YES	NO
Please Explain:	
7. What comments/feedback does your Project student have regarding the leaflet?	
Please Explain:	
